# Anti-oncogenic effects of dutasteride, a dual 5-alpha reductase inhibitor and a drug for benign prostate hyperplasia, in bladder cancer

**DOI:** 10.1186/s12967-023-03972-4

**Published:** 2023-02-18

**Authors:** Jaekwon Seok, Hee Jeong Kwak, Yeonjoo Kwak, Moonjung Lee, Kyoung Sik Park, Aram Kim, Ssang-Goo Cho

**Affiliations:** 1 Department of Stem Cell and Regenerative Biotechnology, Molecular & Cellular Reprogramming Center (MCRC), and Incurable Disease Animal Model & Stem Cell Institute (IDASI), Konkuk University, Seoul, 05029 Republic of Korea; 2 Department of Urology, Konkuk University Medical Center, Konkuk University School of Medicine, Seoul, 05030 Republic of Korea; 3 Department of Surgery, Konkuk University Medical Center, Konkuk University School of Medicine, Seoul, 05030 Republic of Korea; 4 Department of Advanced Translational Medicine, Konkuk University, Seoul, 05029 Republic of Korea

**Keywords:** Dutasteride, Bladder cancer, Androgen receptor, SLC39A9, SRD5A1

## Abstract

**Background:**

The incidence of bladder cancer (BCa) is approximately four times higher in men than in women. To develop effective BCa treatments, there is an urgent need to understand the differences in the BCa control mechanisms based on gender. Our recent clinical study showed that androgen suppression therapy using 5α-reductase inhibitors and androgen deprivation therapy affects BCa progression, but the underlying mechanisms are still unknown.

**Methods:**

mRNA expression levels of the androgen receptor (AR) and SLC39A9 (membrane AR) in T24 and J82 BCa cells were evaluated by reverse transcription-PCR (RT-PCR). The effect of dutasteride, a 5α-reductase inhibitor, in BCa progression was determined in cells transfected with control and AR-overexpressing plasmids. In addition, cell viability and migration assays, RT-PCR, and western blot analysis were performed to analyze the effect of dutasteride on BCa in the presence of testosterone. Finally, steroidal 5α-reductase 1 (*SRD5A1*), one of the dutasteride target genes, was silenced in T24 and J82 BCa cells using control and shRNA-containing plasmids, and the oncogenic role of *SRD5A1* was evaluated.

**Results:**

Dutasteride treatment led to significant inhibition of the testosterone-induced increase dependent on AR and SLC39A9 in cell viability and migration of T24 and J82 BCa cells and induced alterations in the expression level of cancer progression proteins, such as metalloproteases, p21, BCL-2, NF-KB, and WNT in AR-negative BCa. Furthermore, the bioinformatic analysis showed that mRNA expression levels of *SRD5A1* were significantly higher in BCa tissues than in normal paired tissues. A positive correlation between *SRD5A1* expression and poor patient survival was observed in patients with BCa. Also, Dutasteride treatment reduced cell proliferation and migration via blocking the SRD5A1 in BCa.

**Conclusions:**

Dutasteride inhibited testosterone-induced BCa progression dependent on SLC39A9 in AR-negative BCa and repressed oncogenic signaling pathways, including those of metalloproteases, p21, BCL-2, NF-KB, and WNT. Our results also suggest that *SRD5A1* plays a pro-oncogenic role in BCa. This work provides potential therapeutic targets for the treatment of BCa.

**Supplementary Information:**

The online version contains supplementary material available at 10.1186/s12967-023-03972-4.

## Introduction

Bladder cancer (BCa) is a leading cause of death and the 10th most common form of cancer [1]. Worldwide, an estimated 0.5 million new BCa cases and 0.2 million deaths occurred in 2020 [2]. BCa is commonly classified into several types, including non-muscle-invasive BC (NMIBC), muscle-invasive BC (MIBC), and metastatic disease [3]. Approximately 80% of patients with BCa are diagnosed as NMIBC and can be effectively treated with transurethral resection of bladder tumor [4, 5]. However, the recurrence rate is approximately 60%, and up to 45% progress to MIBC [6]. In addition, there is a sex difference in the incidence of BCa, approximately 4:1 male to female ratio, and the mortality rate is also higher in males [7]. Therefore, etiological approaches for advanced BCa therapies are being investigated for effective clinical treatment [3].

Although previously the risk factors for BCa recurrence and progression have been regarded as several environmental elements, such as age, smoking, and sex [3], recent studies suggest that hormonal mechanisms may affect BCa tumorigenesis and progression in vitro and in vivo [8, 9]. Testosterone is an essential sex hormone for the development of male sexual systems and functions [10]. Testosterone is metabolized to a more potent androgen, dihydrotestosterone (DHT), by the enzyme 5α-reductase, which has two isoforms, Type 1 and Type 2 [11]. Recently, DHT has been reported to increase the risk of BCa by interacting with the nuclear and membrane androgen receptor (AR) in vitro and in vivo [9, 12], suggesting that it is an essential factor for the development of BCa and a candidate as a therapeutic target. In addition, clinical studies have been performed to investigate the effect of androgen suppression therapy using 5α-reductase inhibitors or androgen deprivation therapy (ADT) [13–15]. Currently, 5α-reductase inhibitors are used for the treatment of benign prostatic hyperplasia [16]. However, the molecular mechanisms of the effect of 5α-reductase inhibitors in BCa have not been comprehensively studied in vitro and in vivo.

In this study, we used a lentiviral system to overexpress AR and determined the effects of 5α-reductase inhibition in BCa progression induced by testosterone-AR interaction. We analyzed the anti-oncogenic effects of 5α-reductase inhibition on tumor progression in both control BCa cells and AR-overexpressing cells by measuring proliferation, migration, and the expression levels of cancer-related signaling pathways and target genes. In addition, the mRNA expression of steroidal 5α-reductase 1 (*SRD5A1*), a dutasteride target gene, was evaluated in BCa tissues compared to that in normal tissues, and a correlation analysis between SRD5A1 and survival rate was performed using datasets of BCa patients. To validate the clinical observations, lentiviral shRNA for *SRD5A1* was used to knockdown *SRD5A1* expression in vitro, and the effect on progression and migration of BCa cells was evaluated.

## Methods

### Cell lines and treatments

Two human urothelial carcinoma cell lines, T24 and J82, were obtained from the Korea Cell Line Bank (Seoul, Korea) and maintained in RPMI 1640 medium (Sigma-Aldrich, MO, USA), containing 10% fetal bovine serum (FBS) (Gibco, NY, USA) and 1% penicillin/streptomycin (Gibco). The human normal urothelial cell line SV-HUC1 was obtained from the American Type Culture Collection (ATCC, Manassas, USA) and cultured in Nutrient Mixture F-12 K medium (Gibco, NY, USA) supplemented with 10% FBS and 1% penicillin/streptomycin. Cells were cultured in a humidified atmosphere of 5% CO_2_ at 37 °C.

For treatment with 5α-reductase inhibitor and testosterone, T24 and J82 cell lines were cultured with phenol-red-free RPMI 1640 medium (Gibco) containing 10% charcoal striped-FBS (CS-FBS) for 48 h. Dutasteride was selected among a group of 5α-reductase inhibitors because of its dual 5α-reductase inhibition effect. The optimal concentration of 5α-reductase inhibitor was determined by evaluating harmless cytotoxic conditions in normal bladder and BCa cell lines using concentrations ranging from 0 to 5 μM.

### Reverse transcription-polymerase chain reaction and quantitative real-time polymerase chain reaction (qPCR)

Total RNA was extracted using Labozol (CMRZ001, Labopass, Seoul, Korea) according to the manufacturer's instructions. RNA concentration was measured using a NanoPhotometer (IMPLEN, Müchen, Germany), and 2 µg of total RNA was used to synthesized cDNA using the oligo dT primer with M-MuLV reverse transcription kit (CMRT010, Labopass). The cDNA sequences of interest were amplified by PCR using rTaq Plus 5× PCR Master Mix (EBT-1319, Elpisbiotech, Daejeon, Korea) and the primers listed in Additional file 1: Table S1, and the PCR products were analyzed with 1–2% agarose gel electrophoresis. Band densities were measured using Image J (version 1.52p, National Institutes of Health, MD, USA) and normalized against the housekeeping gene GAPDH to calculate relative RNA expression.

PCR products were quantified by quantitative real-time PCR (qPCR) using HiPi Real-Time PCR 2× Master Mix (SYBR green, ROX) (EBT-1802, Elpisbiotech). The primer sequences for the genes analyzed in this study are listed in Additional file 1: Table S2.

### Lentivirus production

The lentiviral plasmid for AR overexpression (pAR) and lentiviral control plasmid (pLenti) were purchased from Addgene (Watertown, MA, USA). Lentiviral packaging and envelope plasmids (Addgene) were used to produce lentiviruses from the packaging cells using Invitrogen Lipofectamine 3000 reagent (Carlsbad, CA, USA). HEK293T cells were cultured in DMEM high medium (Sigma-Aldrich, MO, USA) supplemented with 10% fetal bovine serum and transfected with the viral packaging plasmids and specific lentiviral vector in 6well plate according to the manufacturer’s instructions. After 12–16 h of transfection, culture media were replaced with fresh media and cells were cultured for 48–72 h. Culture media were collected and passed through a syringe filter with a 0.45 µm pore size to obtain the lentiviral soup utilized for infection of the T24 cell line.

To evaluate the SRD5A1-silencing effects in T24 and J82 cell lines, an additional lentiviral plasmid (shSRD5A1) was purchased from Vectorbuilder Inc. (Chicago, USA) and lentiviral samples were prepared as explained above using the same packaging and envelop plasmids.

### Western blot

Total cell lysates were isolated using RIPA lysis buffer (Biosesang, R2002), incubated on ice for 15 min, vortexed every 3 min, and centrifuged at 13,000 rpm for 15 min. Protein concentration in the supernatant was estimated using a BCA protein assay kit (Thermofisher Scientific). Protein samples (10 µg) were separated by electrophoresis using Bolt™ 4–12% Bis–Tris Plus 15-well gels (Invitrogen, NW04125BOX, CA, USA) and transferred to nitrocellulose membranes using iBolt2 Transfer Stacks (Invitrogen, IB23001) and iBolt 2 Dry Blotting System (Invitrogen). Next, the membranes were incubated with primary antibodies against anti-β-actin (1:1000, Santa Cruz, sc-47778), Bcl-2 (1:500, Santa Cruz, sc-509, TX, USA), MMP2 (1:500, Santa Cruz, sc-13595), MMP9 (1:500, Santa Cruz, sc-13520), IκBα (1:500, Cell Signaling Technology, 4814S, Danvers, MA,), phosphorylated-IκBα (1:500, Cell Signaling Technology, 2859S), p65 (1:500, Cell Signaling Technology, 8242S), and phosphorylated-p65 (1:500, Cell Signaling Technology, 3033S) at 4 °C overnight. The membranes were washed three times for 10 min using Tris-buffered saline and Tween 20 buffer (TBS-T; 1:1000) and incubated with secondary antibodies conjugated to horseradish peroxidase (HRP) including anti-mouse (1:3000, Santa Cruz, sc-2005), rabbit (1:3000, Santa Cruz, sc-2004), or goat (1:3000, Santa Cruz, sc-2020) at RT for 2 h. Membranes were then washed with TBS-T for 30 min and the immunoreactive proteins were visualized using an ECL detection kit (Advanstar, K-12045-D50) and measured using iBright Analysis Software (Invitrogen).

### Cell growth and viability assay

The 5α-reductase inhibitor dutasteride (Santa Cruz, sc-207600) was selected for the experiments because it inhibits both the Type-1 and Type-2 isoforms. SV-HUC1, T24, and J82 cell lines were seeded in 12-well culture plates to analyze cell proliferation. The culture medium was replaced with fresh medium containing different dutasteride concentrations (0, 0.01, 0.1, 0.1, 2.5, and 5 µM) to determine the optimal concentration for the experiments. After 24 and 48 h, the number of cells at each concentration was counted using a hemocytometer via trypan blue exclusion. To evaluate the effects on cell growth of 5α-reductase inhibitor treatment, T24 and J82 were cultured in phenol-red free RPMI1640 (Gibco) containing 10% charcoal striped-FBS (CS-FBS) with or without testosterone (10 nM, Abmole, M6105, USA) and 5α-reductase inhibitor for 4 days, and the number of cells was counted every 24 h.

To measure cell viability, T24 pLenti and pAR cells (2 $\times$ 103 cells/well) were cultured into 96-well culture plates, and 10% (v/v) EZ-cytox (DoGeneBio, Seoul, Korea) was added to the medium after 24 and 48 h. After incubation for 1 h, the absorbance in each well was measured at 450 nm using a Bio-RAD x-MarkTM microplate spectrophotometer (Bio-Rad Laboratories, CA, USA), as previously described [17–20].

### Colony forming unit (CFU) assay

T24 and J82 cells (200 cells/well) were cultured with phenol-red free RPMI1640 containing 10% CS-FBS in 6-well plates and changed every 3 days into fresh media with or without testosterone and dutasteride treatment in a humidified atmosphere of 5% CO_2_ at 37 °C for 2 weeks. Then each well was fixed using 4% paraformaldehyde (PFA) (Biosesang, HP2031, Seoul, Korea) and stained with 0.5% crystal violet (Sigma-Aldrich, V5265). Photographs of the colonies were obtained using a light microscope (ZEISS, Axiovert 40 C). and colony numbers were determined using Image J software, as previously described [20].

### Wound closure assay

Cell lines were precultured in phenol-red free RPMI 1640 containing 10% CS-FBS for 48 h with or without testosterone and dutasteride treatment. Then cells were seeded into 6-well culture plates at approximately 90% confluence and treated with 10 µg/mL of mitomycin for 2 h. The cell monolayer was scratched using the end of a 1000-µL pipette tip, and debris caused by the scratch was completely removed. The wound closure areas in the wells were photographed every 12 h for up to 2 days. The closure area was estimated using TScratch (version 1.0, Swiss Federal Institute of Technology, Zurich, Switzerland) and the closure percentage (%) was calculated, as previously described [17–19].

### Migration assay

To analyze the migratory ability, 5 $\times$ 10^4^ cells in 100 µL serum-free media were placed into the upper chamber with 0.8 µm pore size of a transwell plate (Corning, 3413, NY, USA), and culture media containing serum were filled into the bottom chamber. After incubation for 24 h, transwell plates were fixed using 4% PFA and stained with 0.5% crystal violet. Photographs were obtained using a a light microscope (ZEISS, Axiovert 40C). and analyze using ImageJ to estimate the relative migrated area, as previously described [21].

### Clinical expression and survival dataset

The mRNA microarray data of steroid 5 alpha reductase 1, 2 (SRD5A1 and SRD5A2) in BCa and normal tissues were acquired from the Oncomine database version 4.5 (Thermo Fisher Scientific Inc., MI, USA) [22, 23] and analyzed using the Gene Expression Profiling Interactive Analysis (GEPIA, Beijing, China) [24]. Differences in mRNA expression of paired bladder tumor and corresponding normal bladder tissues were considered statistically significant at *p*-value < 0.05 and fold-change cutoff > 2.

The R2: Genomics Analysis and Visualization Platform (Academic Medical Center, Amsterdam, The Netherlands) was used to analyze the survival rate of BCa patients based on SRD5A1 and SRD5A2 mRNA expression. A threshold with a Cox *p*-value < 0.05 was considered statistically significant.

### Gene correlation analysis

Correlated genes of the *SRD5A1* were investigated from TCGA BLCA and Hoglund datasets, using the R2 database. The analysis was performed with the adjustment of the Bonferroni test using a threshold p-value < 0.01. Subsequently, Venn diagrams were used to classify positively correlated genes between TCGA BLCA and Hoglund datasets in BCa, using Venny 2.1.0 (Spanish National Biotechnology Centre (CNB)-CSIC, Madrid, Spain).

### Enrichr gene ontology and signaling pathway analysis

To analyze the ontology and signaling pathway of *SRD5A1* and co-expressed genes, the Enrichr database was used [25]. GO and pathway analyses were visualized as a bar graph. Kyoto Encyclopedia of Genes and Genomes information (KEGG) pathway, Reactome pathway, GO biological process, molecular function, and cellular component were also included in the analysis.

### Statistical analysis

All statistical analyses were performed with GraphPad Prism 7.0 software (GraphPad, La Jolla, CA, USA). Statistical significance was analyzed by multiple *t*-test, one-way ANOVA, and two-way ANOVA. p < 0.05 was considered to be statistically significance.

## Results

### Dutasteride, a 5α-reductase inhibitor, decreases testosterone-AR-induced cell viability and migration

**Fig. 1:**
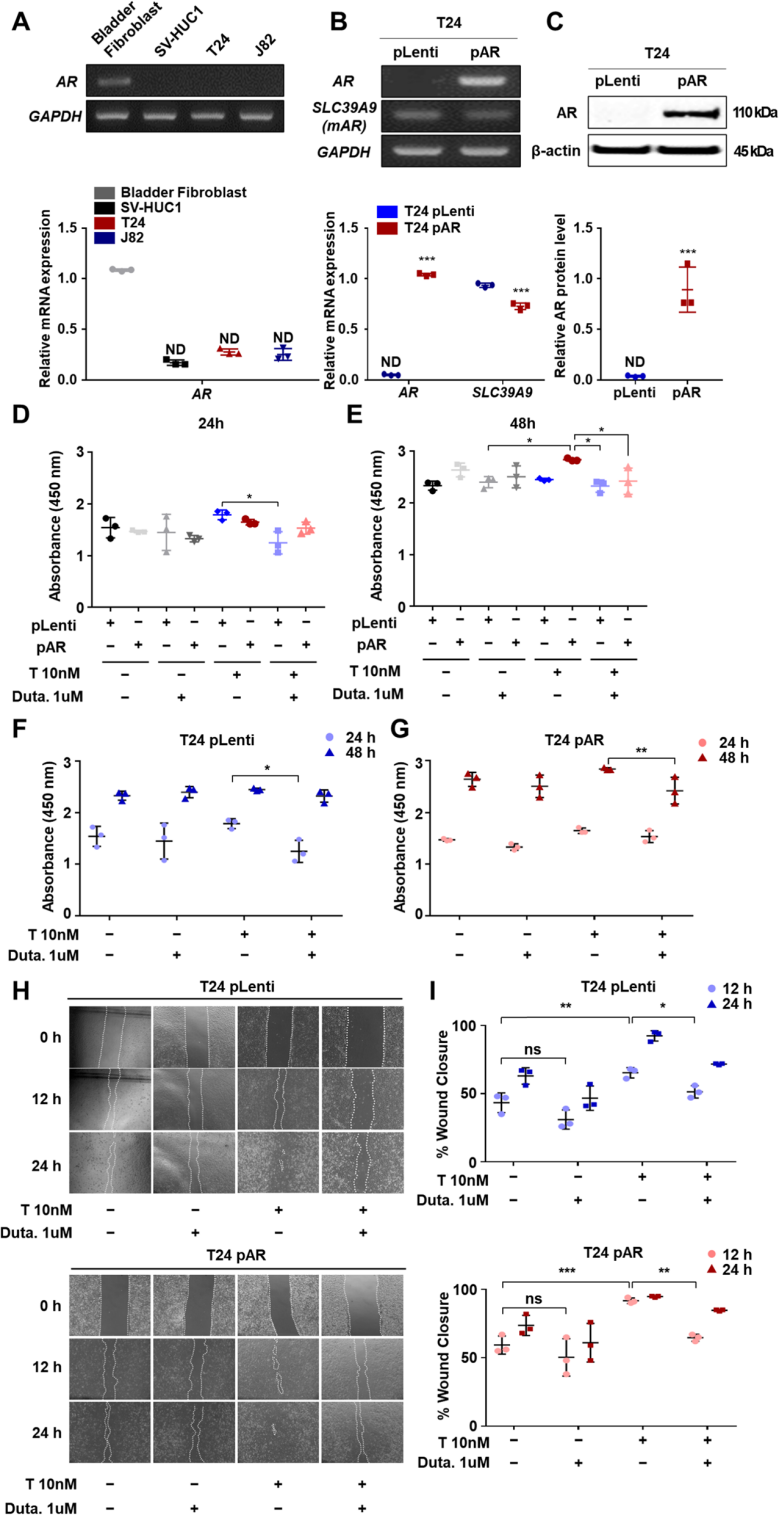
Androgen receptor expression and overexpression in bladder cancer cell lines **A** Representative image of an agarose gel after PCR amplification of the androgen receptor (AR) cDNA synthesized from RNA obtained from normal bladder and cancer cell lines. The graph shows the relative expression level of AR normalized against the housekeeping gene, GAPDH. Number of replicates (N) = 3. **B** Representative image of an agarose gel after PCR amplification of AR and SLC39A9 after lentiviral overexpression of AR in T24 cells. The graph shows the relative expression level normalized against GAPDH. N = 3. **C** Immunoblot probed against AR and β-actin from transduced T24 cells and graph showing the relative protein expression. **D**–**G** Cell viability assay in control (pLenti) and AR-overexpressed T24 cells visualized using EZ-cytox reagent. N = 3. **H** Representative images of the wound closure assay after AR overexpression in transduced-T24 cells. **I** Graphs showing the wound closure percentage measured using T scratch software. N = 3. The statistical analyses were compared using student *t*-test and ordinary two-way ANOVA. All values are estimated as mean ± SD of at least three independent experiments. The statistical significance; *< 0.05, **< 0.01, ***< 0.001, ns > 0.05

To investigate whether the AR affects cancer progression, we examined the mRNA expression of *AR* in various bladder cell lines and observed that *AR* expression was lower in BCa cell lines than in bladder fibroblast cell lines (Fig. 1A, Additional file 1: Fig. S4A). To examine whether dutasteride, a 5α-reductase inhibitor, affected AR-induced cancer progression, we overexpressed AR using an AR lentiviral vector in T24 BCa cells. Then, we measured the mRNA expression of *SLC39A9*, a well-known membrane androgen receptor (mAR), and *AR,* observing clear overexpression after lentiviral transduction (Fig. 1B, Additional file 1: Fig. S4C, D). In addition, the upregulation in AR protein expression was verified by western blot (Fig. 1C). Next, we investigated the effect of the 5α-reductase inhibitor on cell proliferation through cell viability analysis and found that dutasteride inhibited testosterone-AR-induced cell proliferation (Fig. 1D–G). The wound closure assay also showed a significant decrease in closure percentage in T24 pLenti and pAR cells exposed to testosterone and 5α-reductase inhibitor compared to those exposure to testosterone alone (Fig. 1H, I). To confirm that the concentration of dutasteride used in this study did not affect cell survival, SV-HUC1, T24, and J82 cells were treated with increasing concentrations of dutasteride and the number of cells was counted (Additional file 1: Fig. S1). Overall, these results indicate that the 5α-reductase inhibitor prevented AR-induced cell proliferation and migration in BCa cells.

### Inhibition of 5α-reductase decreases testosterone-associated tumor cell proliferation and migration

**Fig. 2:**
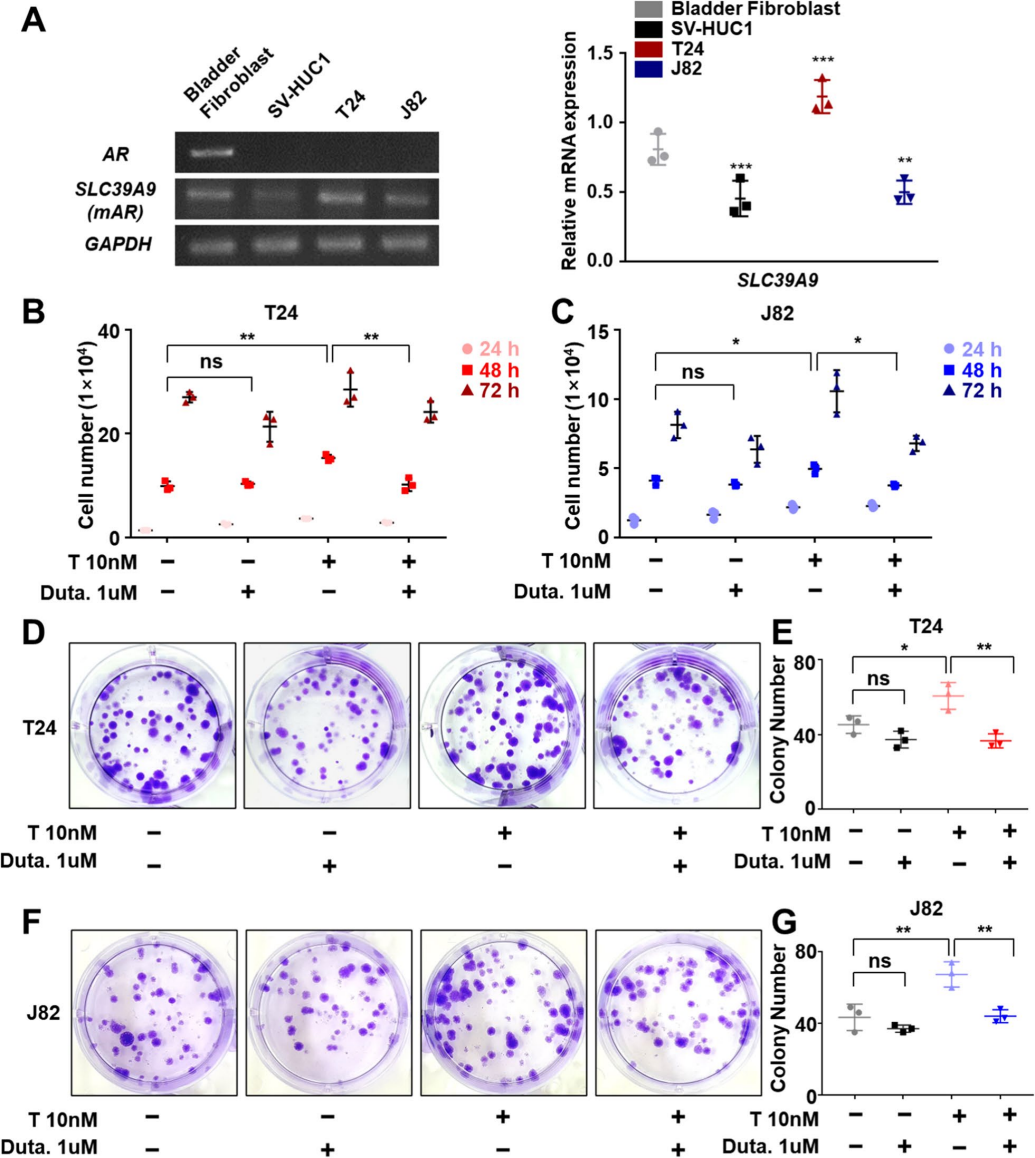
Dutasteride, a 5α-reductase inhibitor, reverses the increase in growth and proliferation of bladder cancer cell lines induced by testosterone treatment **A** mRNA expression analysis of androgen receptor (AR) and SLC39A9 (mAR) in normal bladder and cancer cell lines. The relative expression level was normalized against the housekeeping gene GAPDH. N = 3. **B**, **C** Proliferation of T24 and J82 bladder cancer cells with or without 10 nM testosterone (T) and 1 μM dutasteride was analyzed by counting the number of cells every 24 h for 3 days with trypan blue exclusion. Two-way ANOVA followed by Tukey's multiple comparison tests. N = 3. **D**–**G** Bladder cancer cell colonies were cultured with or without 10 nM testosterone and 1 μM dutasteride and the number of colonies after 2 weeks was quantified. N = 3. One-way ANOVA followed by Tukey’s post-hoc test. All values are shown as mean ± SD of three independent experiments. *p < 0.05, **p < 0.01, ***p < 0.001

To determine the effect of dutasteride in AR-negative cell lines, we evaluated the expression of SLC39A9 (mAR) and observed higher mAR expression in BCa than bladder normal cell lines (Fig. 2A, Additional file 1: Fig. S4B). Several recent studies have indicated that androgens may function via mAR to increase AR-negative cancer progression [9, 26]. Therefore, we evaluated whether the inhibition of testosterone conversion to DHT affects proliferation in BCa cells. Interestingly, the number of surviving T24 and J82 cells significantly increased with 10 nM testosterone treatment and subsequently decreased with co-treatment with 1 μM dutasteride for 24, 48, and 72 h (Fig. 2B, C). To further assess the effects of long-term treatment, we performed a CFU assay and found that testosterone treatment induced an increase in the number of T24 and J82 colonies that was reversed with co-treatment with the 5α-reductase inhibitor (Fig. 2D–G). Interestingly, dutasteride treatment alone did not affect the number of T24 and J82 colonies. Altogether, these results indicate that 5α-reductase inhibition reduces testosterone-induced growth of BCa cell.

**Fig. 3:**
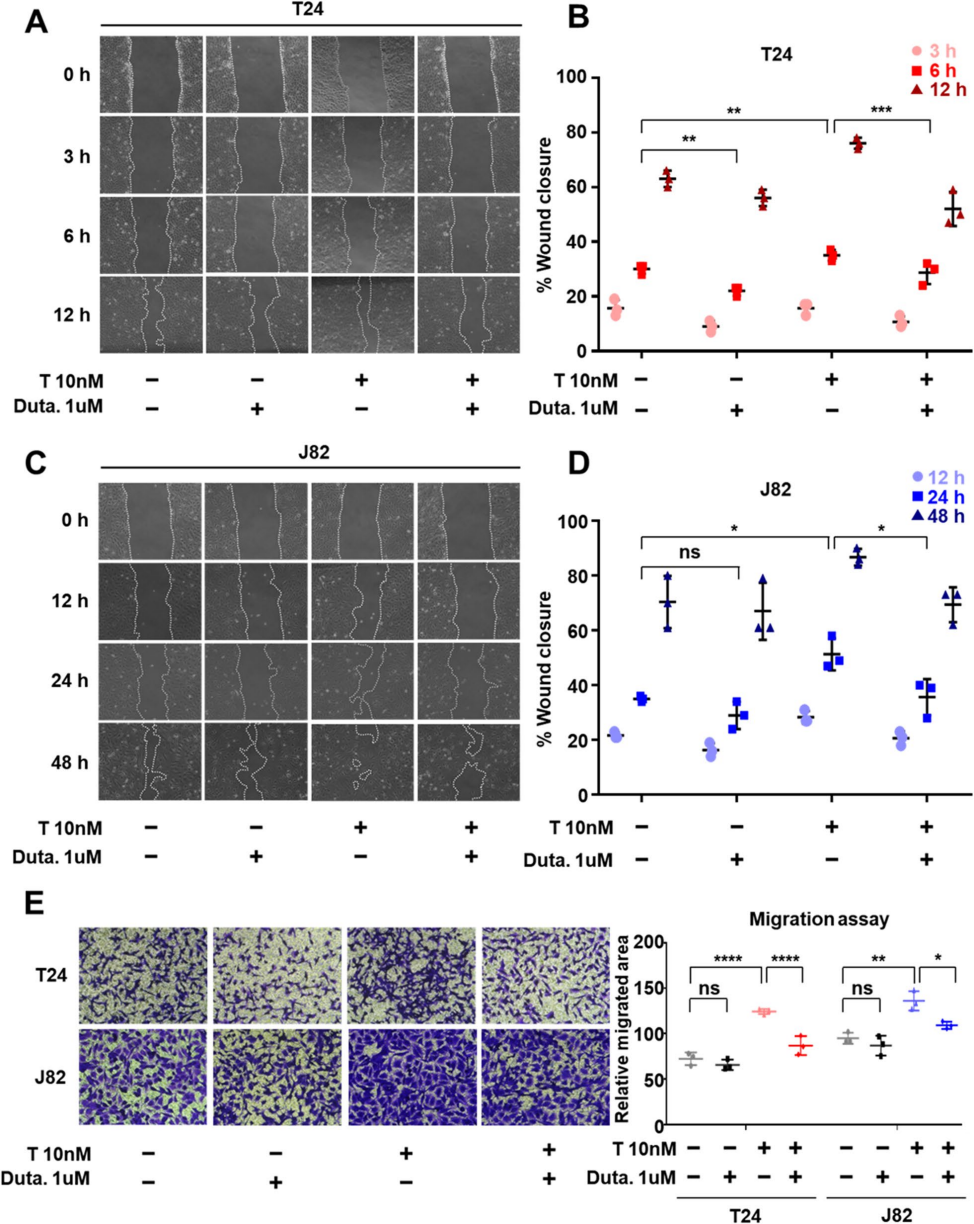
5α-reductase inhibition prevents wound closure and migration of bladder cancer cells induced by testosterone treatment **A**–**D** Wound closure analysis: representative images of T24 (**A**) and J82 (**C**) cells. The graphs on the left indicate the percentage of wound closure with or without testosterone (T) and dutasteride (Duta.) treatments. N = 3. **E** Transwell migration analysis: Images showing the effect of dutasteride treatment on migration and graph showing the relative migrated area. N = 3. All values are shown as mean ± SD of at least three independent experiments, two-way ANOVA in wound closure assay and one-way ANOVA in transwell migration assay followed by Tukey’s post-hoc test. *p < 0.05, **p < 0.01, ***p < 0.001

To examine the effect of 5α-reductase inhibition in metastasis, we performed the tip-scratch and transwell plate in vitro cell migration assays. The results in the tip-scratch assay showed that the closure percentage in T24 and J82 cells increased with testosterone and that co-treatment with dutasteride attenuated the cell migration kinetics (Fig. 3A–D). In agreement, the results of the migration assay in transwell plates indicated that 10 nM testosterone treatment significantly increased T24 and J82 migration, and co-treatment with 1 µM dutasteride significantly suppressed cell migration to the lower chamber (Fig. 3E). Overall, these results indicate that dutasteride can decrease cell proliferation, migration, and invasion abilities on BCa AR-negative cell lines as on BCa AR-positive cell lines.

### 5α-reductase inhibition prevents the increase in protein levels associated with tumor progression induced by testosterone treatment

**Fig. 4:**
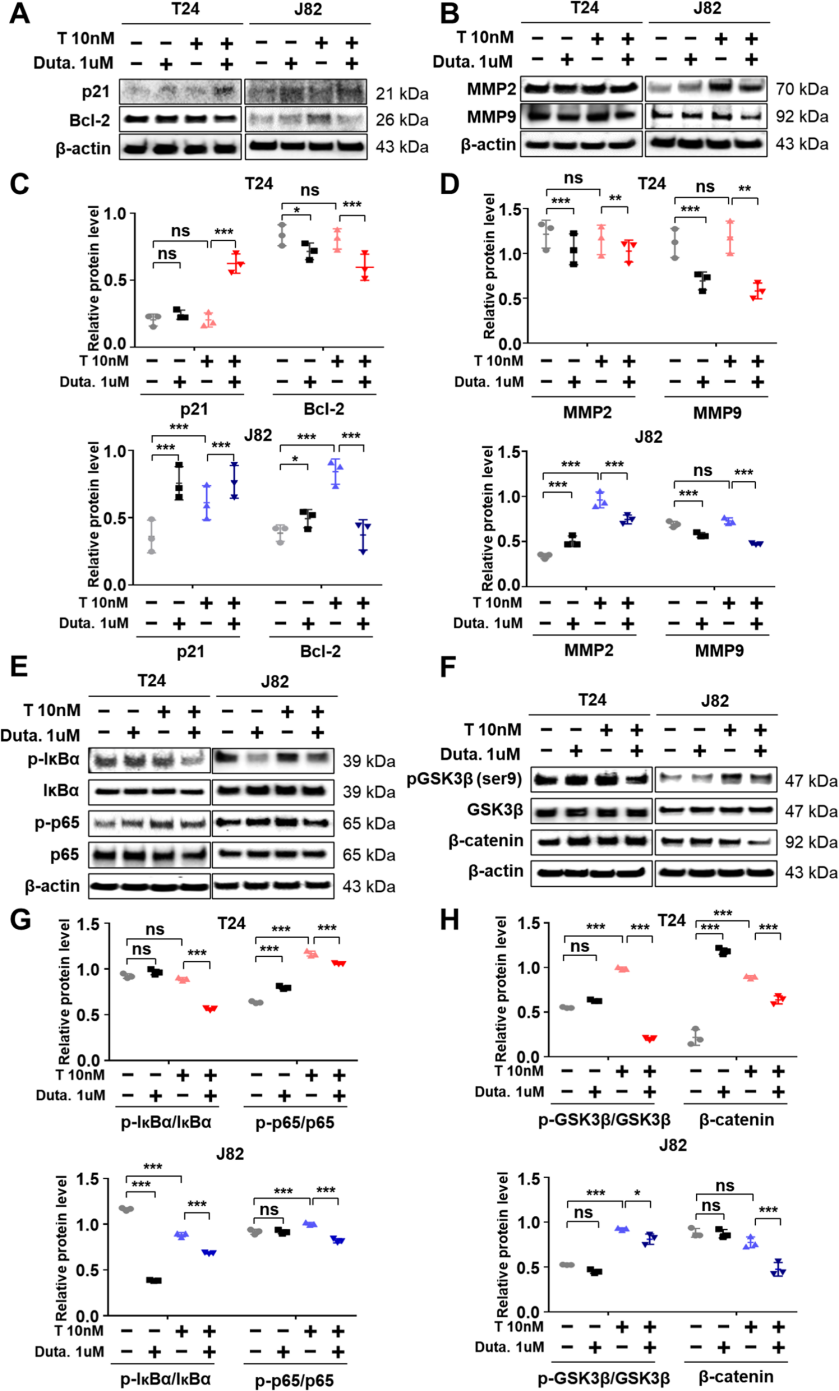
5α-reductase inhibition decreases the translational level of cancer progression-related proteins in bladder cancer cells treated with testosterone **A**, **B** Protein expression levels related to cancer survival and metastasis; p21, Bcl-2, matrix metalloproteinase-2 (MMP2), and MMP9 were analyzed using western blot. β-actin was used as an internal standard for band normalization. Western blot bands were measured and calculated using Image J. N = 3. **C** Graphs showing relative protein expression of Bcl-2 and P21 in T24 and J82 cells. **D** Graphs showing relative protein expression of MMP2 and MMP9 in T24 and J82 cells. **E**, **F** Protein expression levels related to NF-κB and GSK3β signaling pathways analyzed using western blot. β-actin was used as an internal standard for band normalization. Western blot bands were measured and calculated using Image J. N = 3. **G** Graphs showing relative protein expression of phospho-IκBα and phospho-p65 in T24 and J82 cells. Total IκBα and p65 were used as a standard for band normalization. **H** Graphs showing relative protein expression of phospho-GSK3β and β-catenin in T24 and J82 cells. Total GSK3β was used as a standard for band normalization. All values are shown as the mean ± SD of at least three independent experiments, and the statistical significance was analyzed using two-way ANOVA, indicated as *< 0.05, **< 0.01, ***< 0.001

Inhibition of 5α-reductase with dutasteride prevented the increase in cell viability, migration, and proliferation induced by testosterone treatment. To investigate the underlying mechanism, we evaluated several tumor growth and migration markers by western blot, specifically Bcl-2 and p21, modulators of apoptosis and cancer progression [27–29], respectively, and metalloproteinase-2 (MMP2) and -9 (MMP9), related with tumor invasion ability. Testosterone treatment significantly increased the protein levels of pro-survival Bcl-2, whereas the expression of the cell cycle arrest-associated p21 protein decreased (Fig. 4A, C). In addition, the protein levels of MMP2 and MMP9 increased in T24 and J82, respectively, with testosterone treatment (Fig. 4B, D). Interestingly, co-treatment with testosterone and the 5α-reductase inhibitor reduced Bcl-2, MMP2, and MMP9 protein levels and potentiated p21 expression. We further examined several upstream signaling pathways correlated with tumor survival and migration. Phosphorylated IκBα, GSK3β, p65, and β-catenin expression levels significantly decreased in T24 and J82 cells after co-treatment with testosterone and dutasteride compared to those after testosterone treatment alone (Fig. 4E–H). Altogether, these results suggest that 5α-reductase inhibition may decrease cancer cell progression and migration via p21, Bcl-2, MMP2, MMP9, NF-κB, and GSK3β signaling pathways.

### Expression and survival rate of steroidal 5α-reductase 1 (SRD5A1) in BCa

**Fig. 5:**
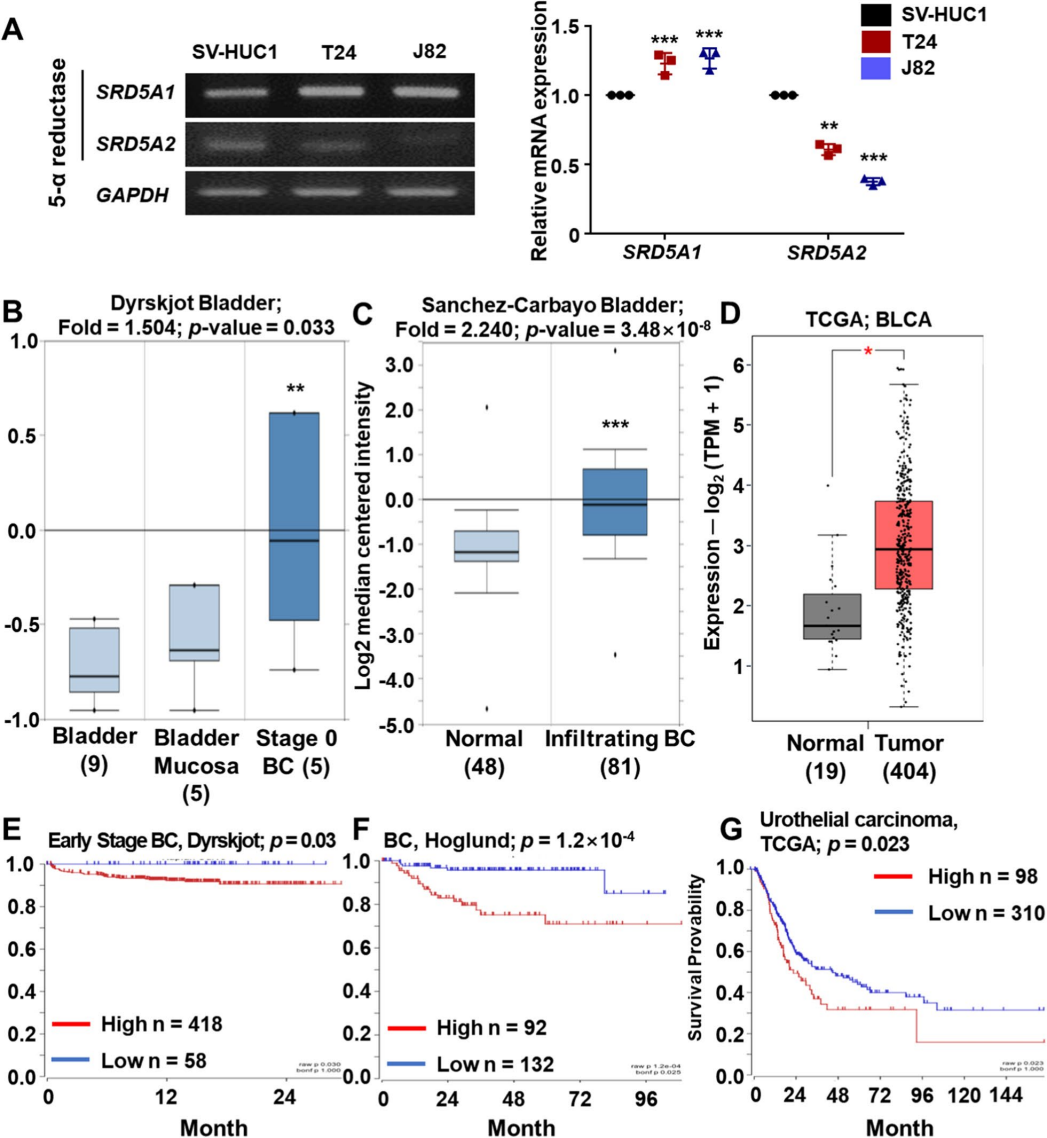
Expression of Steroidal 5α-reductase 1 (*SRD5A1*) and correlation with survival rate in patients with bladder cancer **A** Reverse transcription-polymerase chain reaction (RT-PCR) was used to measure relative *SRD5A1* mRNA expression in the human urothelial cell line, SV-HUC1, and bladder cancer cell lines, T24 and J82, and relative mRNA expression was calculated against glyceraldehyde 3-phosphate dehydrogenase (*GAPDH*). N = 3. **B**–**D** mRNA expression levels of *SRD5A1* in bladder cancer tissues compared with normal tissues. Data were obtained from the Gene Expression Profiling Interactive Analysis (GEPIA) and Oncomine webtool and were statistically determined as fold-change threshold > 1.5 in the Oncomine dataset and *p*-value < 0.05 in all databases. **E**–**G** Survival rate of patients with bladder cancer in groups with high and low *SRD5A1* expression from different datasets obtained from the R2 database. *p*-values are shown in the graphs

The 5α-reductase inhibitor dutasteride inhibits both 5α-reductase Type 1 and 2 [30]. We assessed the mRNA expression levels of SRD5A1 and SRD5A2 in normal human urothelial cells, SV-HUC1 and BCa cell lines, T24 and J82. RT-PCR analysis showed that the mRNA expression level of *SRD5A1* was significantly higher in T24 and J82 than in SV-HUC1 cells, whereas *SRD5A2* expression decreased (Fig. 5A, Additional file 1: Fig. S4E, F). To investigate if those differences were present in patients with BCa, we analyzed the mRNA expression of *SRD5A1* in BCa tissues and normal tissues using public clinical web tools, such as GEPIA and Oncomine. Analysis of the Dyrskjot dataset [31], containing expression data from 14 normal bladder tissues and five stage 0 BCa tissues, and the Sanchez-Carbayo dataset [32], containing 48 normal bladder tissues and 81 infiltrating BCa tissues, showed higher mRNA expression of *SRD5A1* in BCa tissues than in normal tissues (Fig. 5B, C). In addition, we obtained mRNA expression profiles from BCa and adjacent normal tissues from GEPIA (TCGA Data Online Analysis Tool) [33], and observed that *SRD5A1* expression was significantly upregulated in BCa tissues compared to that in normal bladder tissues (Fig. 5D). Interestingly, correlation analysis of the survival curves in patients with BCa and *SRD5A1* mRNA expression levels obtained from the Dyrskjot, Hoglund, and TCGA datasets indicated that high *SRD5A1* mRNA expression was negatively correlated with overall survival (Fig. 5E–G) [33–35], suggesting a tumor-progressive role of *SRD5A1*. However, the mRNA expression level of *SRD5A2* was not different between BCa and normal tissues, and the correlation analysis showed a non-significant effect of high expression of *SRD5A2* on survival (Additional file 1: Fig. S2). Also, we found positively correlated genes with *SRD5A1* from TCGA and Hoglund datasets represented by a Venn diagram using the R2 database (Additional file 1: Fig. S3A). Subsequently, we investigated commonly related pathways of *SRD5A1* and 1798 correlated genes, the pathway analysis were classified in cell cycle, DNA replication, and other pathways. In GO analysis, *SRD5A1* and its positively co-expressed genes were related with mitosis and DNA replication (Additional file 1: Fig. S3B). These results suggest that *SRD5A1* expression may have a strong positive correlation with patient survival and affect many signaling pathway including cell cycle and DNA replication in bladder cancer.

### Silencing of SRD5A1 represses tumor progression in BCa cells

**Fig. 6:**
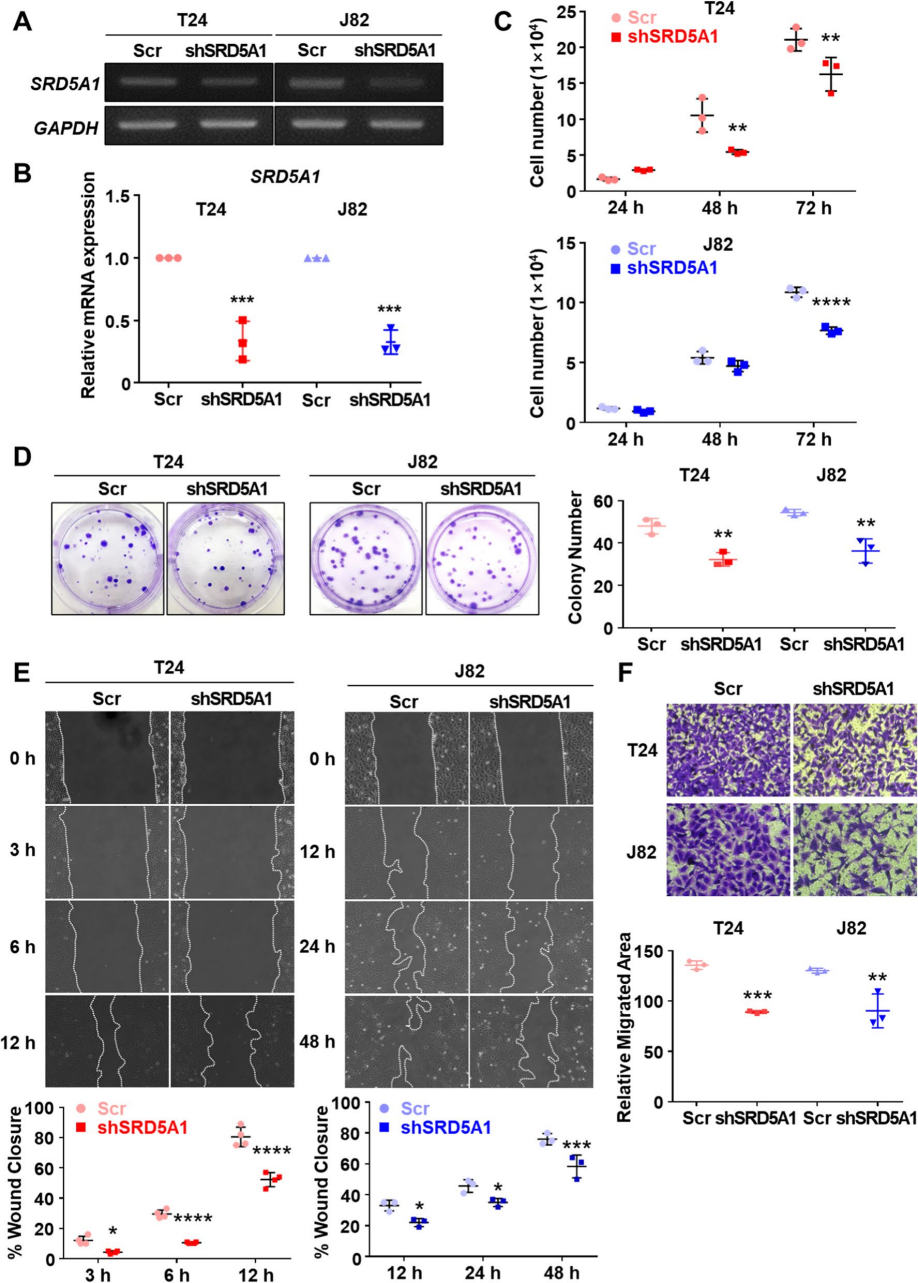
Effects of *SRD5A1* silencing in T24 and J82 bladder cancer cell lines **A**, **B** Gene expression of *SRD5A1* in shSRD5A1-transduced and control (Scr) cells was analyzed using reverse transcription-polymerase chain reaction (RT-PCR). The PCR products were run in an agarose gel (**A**) and the relative mRNA expression of *SRD5A1* was normalized against *GAPDH* (**B**). N = 3. **C** The number of *SRD5A1*-silenced cells and control cells was counted after trypan blue exclusion for 3 days. N = 3. **D** Representative images from the colony forming unit assay and quantification of the number of colonies in shSRD5A1-transduced and control Scr cells. N = 3. **E** Cell closure analysis. The upper panels represent wound closure of control and *SRD5A1-*silenced cells, and the closure rates are indicated in the bottom panel. N^T24^ = 4. N^J82^ = 3 **F** Migration analysis in T24 and J82 control and shSRD5A1 cells using transwell. The upper panels show representative images of the migration analysis and the relative migrated area is shown in the bottom panel. N = 3. All values are expressed as mean ± SD of at least three independent experiments, and statistical significance was analyzed using ordinary one-way ANOVA and two-way ANOVA. (*p < 0.05; **p < 0.01; ***p < 0.001; ****p < 0.0001)

To assess the role of SRD5A1 in BCa cells, we used SRD5A1-targeted shRNA to silence SRD5A1 expression in T24 and J82 cells. We used RT-PCR to confirm the downregulation of SRD5A1 expression and observed a decrease of approximately 70–80% in T24 and J82 cell numbers (Fig. 6A, B, Additional file 1: Fig. S4G, H). We then compared tumor cell growth between control (Scr) and SRD5A1-silenced T24 and J82 cells. The number of living cells significantly decreased in SRD5A1 knockdown cells from day 2 (Fig. 6C). In addition, the CFU assay revealed that the cell proliferation rate was significantly higher in control T24 and J82 cells than in SRD5A1 silenced cells (Fig. 6D). We performed the wound closure and transwell migration in vitro assays to further characterize the oncogenic properties of SRD5A1. The closure rate was lower in SRD5A1-silenced cells, being significant after 12 h and 48 h in T24 and J82 cells, respectively (Fig. 6E). In agreement, the transwell migrated area was significantly reduced in both SRD5A1 knockdown BCa cell lines (Fig. 6F). Overall, these results indicate that knockdown of SRD5A1 attenuates the oncogenic properties of BCa cells, including growth, self-renewal, and migration.

## Discussion

Although improvements in BCa management have been made to enhance patient prognosis, currently there is no definitive strategy to decrease the recurrence rate of BCa and its treatment is continually regarded as an economic burden [36]. In addition, there is no oral medication that can reduce recurrence and progression in patients with BCa. Traditionally, Bacillus Calmette-Guerin (BCG) immunotherapy has been used to decrease the rate of recurrence of patients with NMIBC [37]. However, although BCG therapy is still the most effective strategy to prevent recurrence, several potential side effects have occurred in patients with NMIBC treated with BCG, including high fever, discomfort in the bladder, and urinary tract infection [38, 39]. In addition, in patients that do not respond to immunotherapy, the treatment options are very limited and their recurrence remains dismal.

Efforts have been made to discover alternative treatments for NMIBC with respect to hormone therapy, and to understand the underlying molecular mechanisms related to sex differences, such as androgen hormones and ARs, as BCa has been shown to have a high incidence in males [13, 26, 40]. In this study, we showed that BCa progression was affected by androgen hormones, and 5α-reductase inhibition restrained testosterone-induced proliferation and migration in BCa cells.

Testosterone is converted in DHT by 5α-reductase. DHT has been reported to be more potent than testosterone, and its implications extend to prostate growth and several cancer types, including prostate, lung, colorectal, and bladder cancers [26, 41]. Therefore, 5α-reductase inhibitors have been investigated for the treatment of BCa in males, observing a significantly reduced incidence [15]. In addition, androgen suppression therapy in male patients with NMIBC has been significantly associated with a lower recurrence rate [42]. Despite these well-established clinical evidences, the mechanisms by which 5α-reductase inhibition improves the therapeutic management in BCa have not been clarified yet.

Testosterone and DHT, the main androgens, interact with AR. AR belongs to a nuclear receptor superfamily and it modulates target gene expression via nuclear localization [43]. However, previous studies have revealed the existence of a membrane AR that binds to androgens to activate rapid cell surface steroid physiological actions in prostate, breast, and bladder cancers via Bax, p53, and JNK expression, and MAPK/MMP9 intracellular signaling [9, 44–46]. In this study, we induced AR overexpression, measured the expression levels of *AR* and the membrane AR *SLC39A9*, and evaluated the effects of 5α-reductase inhibition combined with testosterone treatment in BCa cells. These results indicated that 5α-reductase inhibition affects AR-positive and -negative cell lines in the presence of membrane AR and testosterone. Therefore, we focused on the anti-oncogenic effects of 5α-reductase inhibition and evaluated the effects on cancer progression of the dutasteride target genes *SRD5A1* and *SRD5A2*, encoding 5α-reductase Type 1 and 2, respectively. Our results indicated that cancer progression was potentiated by testosterone treatment and attenuated by 5α-reductase inhibition, leading to alterations in expression of cancer protein markers, including Bcl-2, MMP2, and MMP9. A previous study reported that knockdown of *SRD5A1* and 5α-reductase inhibitor treatment in vitro induced autophagy via the PI3K/AKT/mTOR pathway [47], supporting a Bcl-2-regulated signaling mechanism. However, further experiments to measure testosterone and DHT levels after co-treatment with testosterone and dutasteride are required to evaluate the efficiency of the 5α-reductase inhibitor.

Our results suggest that *SRD5A1* is an oncogenic player in BCa, and 5α-reductase inhibition may decrease and control tumor growth in patients with BCa. Bioinformatic analysis of clinical datasets showed a significant increase in *SRD5A1* mRNA expression in BCa tissues that was negatively correlated with the survival probability of patients. Also, we found SRD5A1 and its positively correlated genes were related with cell cycle, DNA replication, and mitosis from various bladder cancer datasets in R2 and Enrichr databases. In contrast, mRNA expression of *SRD5A2* in BCa tissues was not significantly correlated with survival rate. Subsequently, we examined tumor growth and migration in *SRD5A1* knockdown BCa cells and found a significant clinical therapeutic role of 5α-reductase inhibition for the treatment of BCa. In the previous report, genomic alterations in 5α-reductases was found in approximately 30% of the BCa patients, and patients with these alterations had a negative correlation with disease-free survival [48]. Therefore, 5α-reductase inhibitors must play a crucial therapeutic role to suppress cancer development, improve prognosis, and independently modulate AR activity.

However, this study presents a few limitations. First, although the in vitro results obtained in BCa cells and the bioinformatic analysis using various databases of patients with BCa suggest an anti-oncogenic effect of 5α-reductase inhibition, further in vivo experiments using animal models are required to elucidate the underlying mechanisms of 5α-reductase inhibition in BCa. Second, we did not determine the anti-oncogenic effects of finasteride, another 5α-reductase Type 2 inhibitor, in BCa cell lines, and further investigations must be carried out in the future to compare its effects with those of dutasteride.

## Conclusions

This study suggests that the 5α-reductase inhibitor dutasteride may play an anti-oncogenic role in BCa. Specifically, our results revealed that 5α-reductase inhibition attenuate tumor progression by inhibiting the conversion of testosterone to DHT in the presence of membrane AR in BCa. In addition, we showed that dutasteride reduces BCa progression by blocking SRD5A1, one of the target proteins of the 5α-reductase inhibitor. Taken together, these results suggest that 5α-reductase inhibitors may constitute potential therapeutic agents for the treatment of BCa.

## Supplementary Information

**Additional file 1: Figure S1.** Effect of dutasteride treatment on normal bladder cell line and bladder cancer cell lines. Graphs show the number of normal urothelial cells (SV-HUC1) and bladder cancer cells (T24 and J82) after treatment with different concentrations of dutasteride ranging from 0 to 5 μM. (ns: no significant difference, *: *p* < 0.05, **p < 0.01, ***: *p* < 0.001). **Figure S2.** SRD5A2 expression and patient survival plot in patients with bladder cancer. **(A-C)** The mRNA expression levels of *SRD5A2* are not significantly different between bladder cancer tissue and the normal tissue counterparts in various datasets. Data were obtained from the Gene Expression Profiling Interactive Analysis (GEPIA) and Oncomine webtool and were statistically determined as fold-change threshold > 1.5 in the Oncomine dataset and p-value threshold < 0.05 in all databases. **(D-F)** Survival rate of patients with bladder cancer in high and low SRD5A2 expression groups from the R2 database. Statistical significance was used *p*-value threshold < 0.05. **Figure S3.** Signaling pathways of *SRD5A1* related with cancer proliferation in urothelial carcinoma. **(A)** Venn diagram of the genes positively correlated with *SRD5A1*, generated from the TCGA-BLCA and Hoglund transcriptome dataset using the R2 database. **(B)** GO and pathway analysis with *SRD5A1* and co-expressed genes using Enrichr database; bar graph listed by *p*-value. The brighter the bar color, the more significant the related pathway. **Figure S4.** Gene expression analysis of various bladder cell lines. Quantitative polymerase chain reaction analysis of the gene encoding androgen receptor **(A, C)**, solute carrier family 39 member 9 **(B, D)**, steroid 5 alpha-reductase 1, 2 **(E–H)** in the indicated bladder normal and cancer cell lines. The statistical analyses were compared using student t-test and ordinary two-way ANOVA. All values are estimated as mean ± SD of at three independent experiments. (*: *p* < 0.05, **p < 0.01, ***: *p* < 0.001, ****: *p* < 0.0001). **Table S1.** Oligonucleotides used for RT-PCR. **Table S2.** Oligonucleotides used for RT-qPCR

## Data Availability

All data generated or analyzed during this study are included in this published article and its additional files.

## Abbreviations

BCa: Bladder cancer
AR: Androgen receptor
SLC39A9: Solute carrier family 39 member 9
SRD5A1: Steroidal 5α-reductase 1
SRD5A2: Steroidal 5α-reductase 2
NMIBC: Non-muscle-invasive BC
MIBC: Muscle-invasive BC
DHT: Dihydrotestosterone
GEPIA: Gene expression profiling interactive analysis
shRNA: Short hairpin RNA
