# Supplementary material for: Anti-oncogenic effects of dutasteride, a dual 5-alpha reductase inhibitor and a drug for benign prostate hyperplasia, in bladder cancer
Source: J Transl Med. 2023 Feb 18;21:129. doi: 10.1186/s12967-023-03972-4 (PMC9938606; doi:10.1186/s12967-023-03972-4)
Supplement: Supplementary file 1 — Additional file 1: Figure S1. Effect of dutasteride treatment on normal bladder cell line and bladder cancer cell lines. Graphs show the number of normal urothelial cells (SV-HUC1) and bladder cancer cells (T24 and J82) after treatment with different concentrations of dutasteride ranging from 0 to 5 μM. (ns: no significant difference, *: p < 0.05, **p < 0.01, ***: p < 0.001). Figure S2. SRD5A2 expression and patient survival plot in patients with bladder cancer. (A-C) The mRNA expression levels of SRD5A2 are not significantly different between bladder cancer tissue and the normal tissue counterparts in various datasets. Data were obtained from the Gene Expression Profiling Interactive Analysis (GEPIA) and Oncomine webtool and were statistically determined as fold-change threshold > 1.5 in the Oncomine dataset and p-value threshold < 0.05 in all databases. (D-F) Survival rate of patients with bladder cancer in high and low SRD5A2 expression groups from the R2 database. Statistical significance was used p-value threshold < 0.05. Figure S3. Signaling pathways of SRD5A1 related with cancer proliferation in urothelial carcinoma. (A) Venn diagram of the genes positively correlated with SRD5A1, generated from the TCGA-BLCA and Hoglund transcriptome dataset using the R2 database. (B) GO and pathway analysis with SRD5A1 and co-expressed genes using Enrichr database; bar graph listed by p-value. The brighter the bar color, the more significant the related pathway. Figure S4. Gene expression analysis of various bladder cell lines. Quantitative polymerase chain reaction analysis of the gene encoding androgen receptor (A, C), solute carrier family 39 member 9 (B, D), steroid 5 alpha-reductase 1, 2 (E–H) in the indicated bladder normal and cancer cell lines. The statistical analyses were compared using student t-test and ordinary two-way ANOVA. All values are estimated as mean ± SD of at three independent experiments. (*: p < 0.05, **p < 0.01, ***: p < 0.001, ****: p < 0.0001). T [file 12967_2023_3972_MOESM1_ESM.docx]

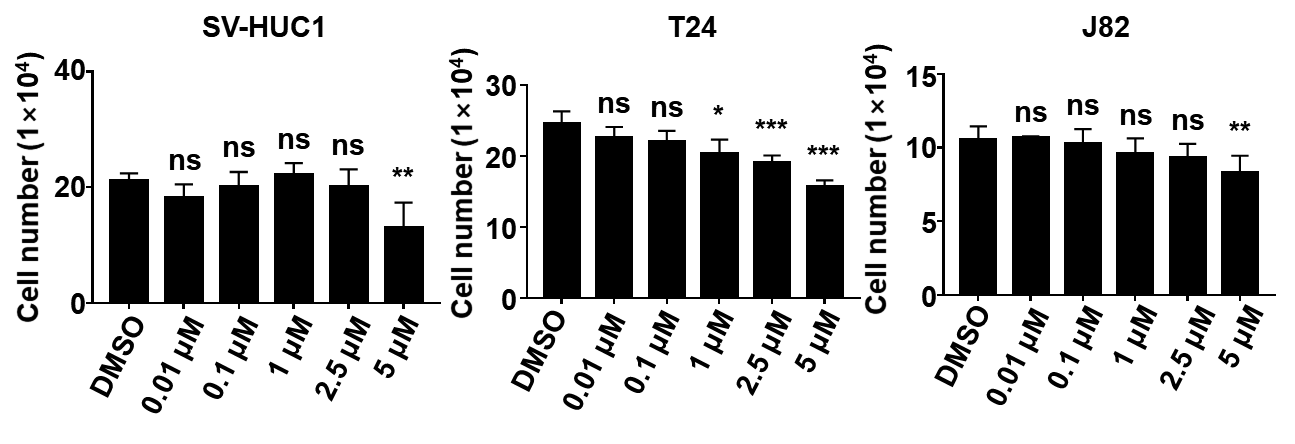


**Fig. S1. Effect of dutasteride treatment on normal bladder cell line and bladder cancer cell lines** Graphs show the number of normal urothelial cells (SV-HUC1) and bladder cancer cells (T24 and J82) after treatment with different concentrations of dutasteride ranging from 0 to 5 μM. (ns: no significant difference, *: *p* < 0.05, ***p* < 0.01, ***: *p* < 0.001)


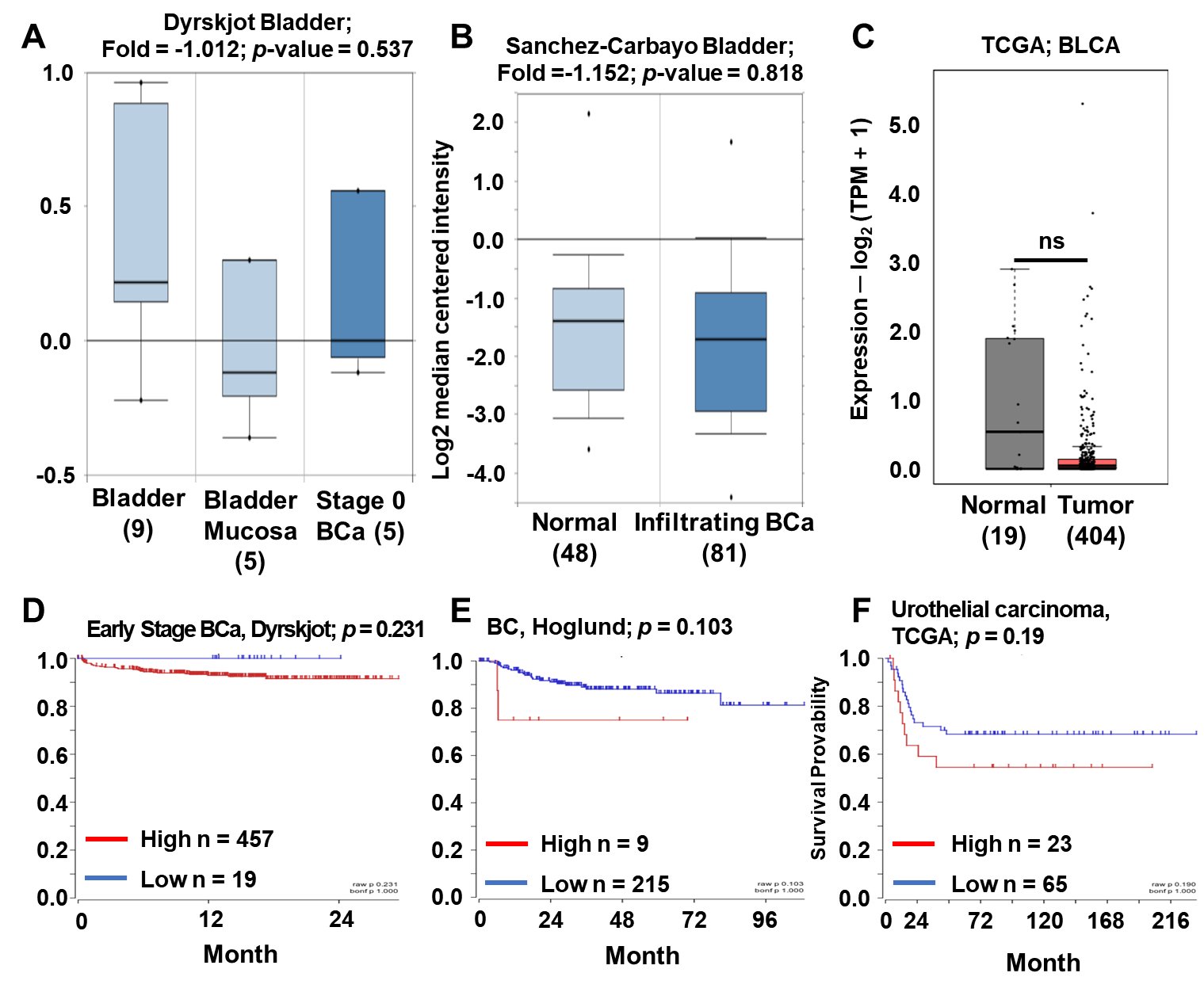


**Fig. S2. SRD5A2 expression and patient survival plot in patients with bladder cancer (A-C)** The mRNA expression levels of *SRD5A2* are not significantly different between bladder cancer tissue and the normal tissue counterparts in various datasets. Data were obtained from the Gene Expression Profiling Interactive Analysis (GEPIA) and Oncomine webtool and were statistically determined as fold-change threshold > 1.5 in the Oncomine dataset and p-value threshold < 0.05 in all databases. **(D-F)** Survival rate of patients with bladder cancer in high and low SRD5A2 expression groups from the R2 database. Statistical significance was used *p*-value threshold < 0.05.

**
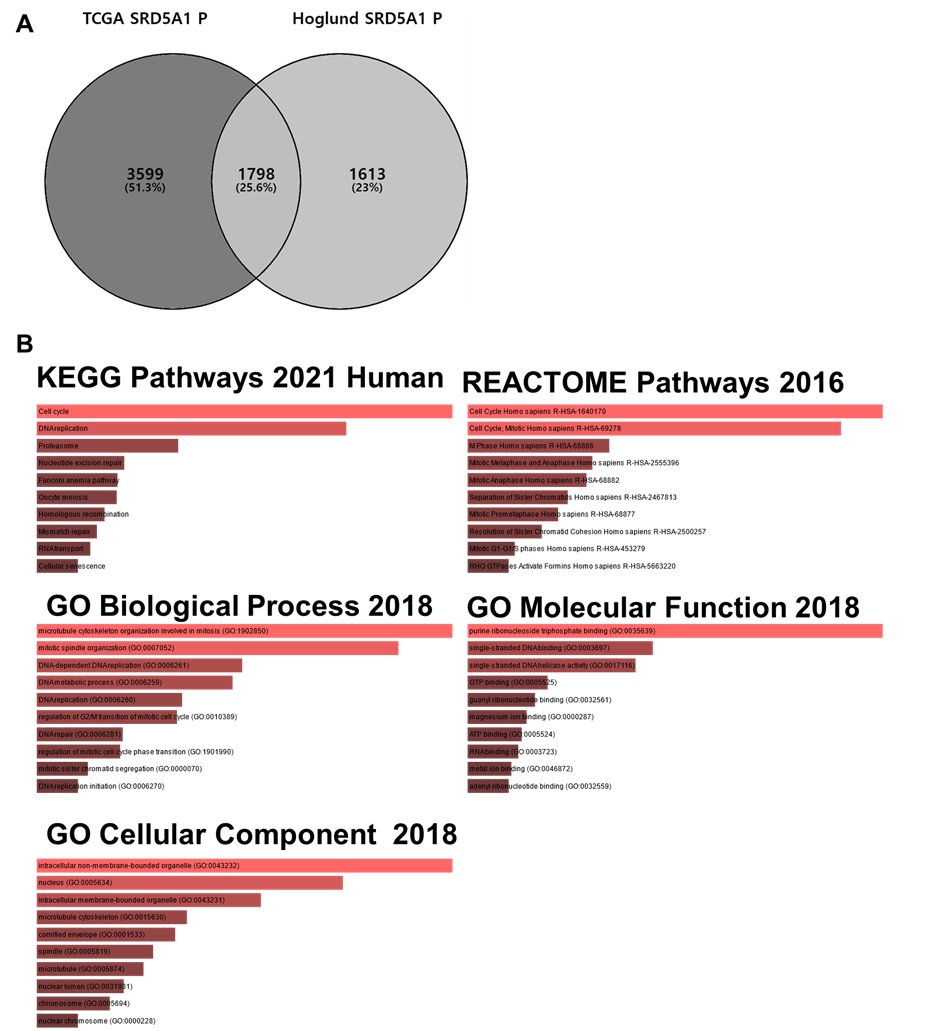
**

**Fig. S3. Signaling pathways of *SRD5A1* related with cancer proliferation in urothelial carcinoma (A)** Venn diagram of the genes positively correlated with *SRD5A1*, generated from the TCGA-BLCA and Hoglund transcriptome dataset using the R2 database. **(B)** GO and pathway analysis with *SRD5A1* and co-expressed genes using Enrichr database; bar graph listed by *p*-value. The brighter the bar color, the more significant the related pathway.

**
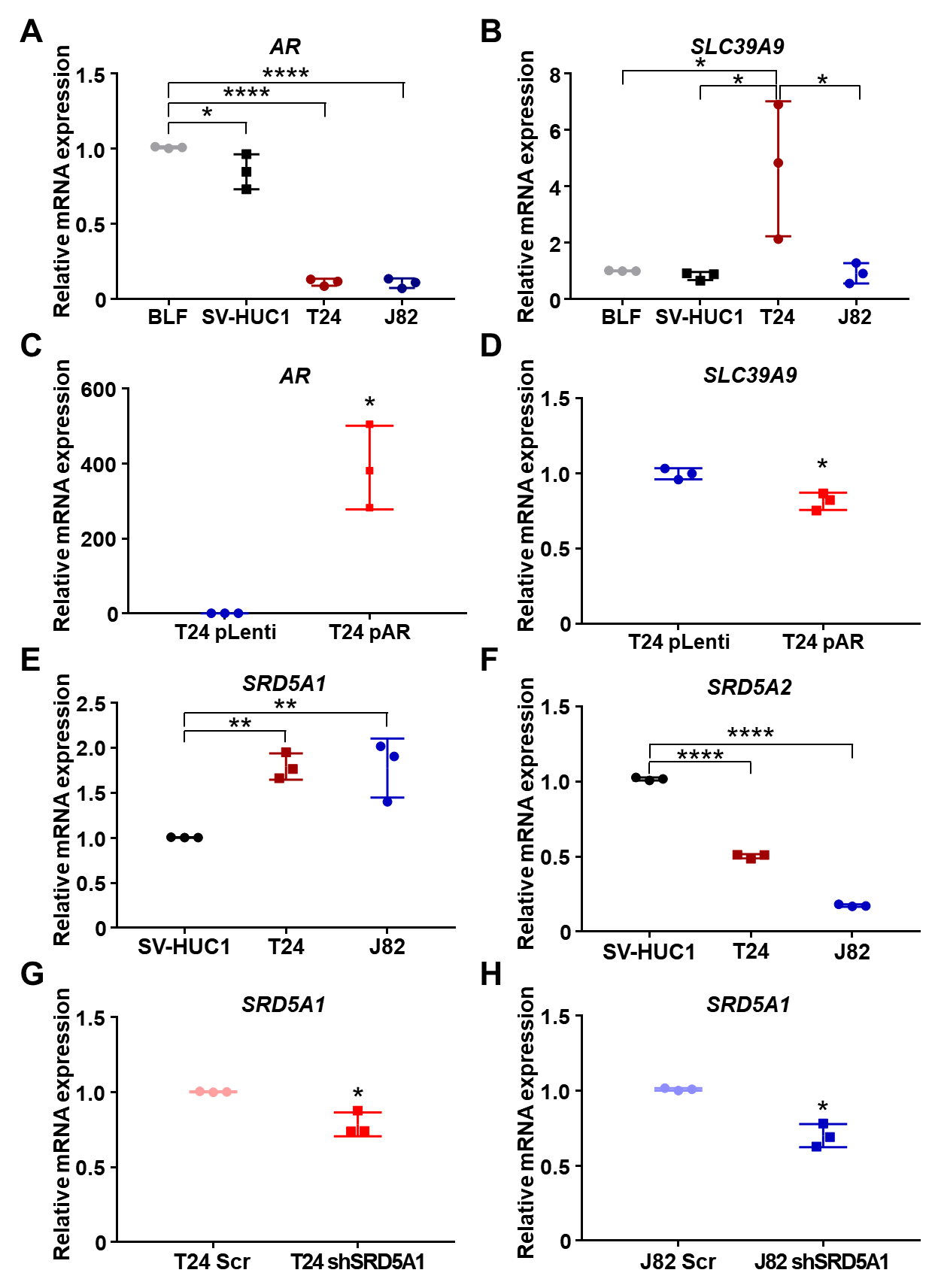
**

**Fig. S4. Gene expression analysis of various bladder cell lines** Quantitative polymerase chain reaction analysis of the gene encoding androgen receptor **(A, C)**, solute carrier family 39 member 9 **(B, D)**, steroid 5 alpha-reductase 1, 2 **(E-H)** in the indicated bladder normal and cancer cell lines. The statistical analyses were compared using student t-test and ordinary two-way ANOVA. All values are estimated as mean ± SD of at three independent experiments. (*: *p* < 0.05, ***p* < 0.01, ***: *p* < 0.001, ****: *p* < 0.0001)

**Table S1. Oligonucleotides used for RT-PCR**

| **Oligo** | **Forward Sequence (5' to 3')** | **Reverse Sequence (5' to 3')** | **Size** | **Tm (℃)** |
| --- | --- | --- | --- | --- |
| hGAPDH | AAT CCC ATC ACC ATC TTC CAG | CAC GAT ACC AAA GTT GTC ATG | 300 | 58 |
| hAR | AAG GAA CTC GAT CGT ATC ATT GC | AAT AGA TGG GCT TGA CTT TCC CA | 241 | 59 |
| hSLC39A9 | TGG TGA CTG TTT TGG GTG CT | TGC TTG GTG GTG TTT TCC CT | 113 | 60 |
| hSRD5A1 | GGG TAA CAG ATC CCC GTT TT | AGC CAC ACC ACT CCA TGA TT | 192 | 58 |
| hSRD5A2 | CAT ACG GTT TAG CTT GGG TGT | GCT TTC CGA GAT TTG GGG TAG | 315 | 60 |

**Table S2. Oligonucleotides used for RT-qPCR**

| **Oligo** | **Forward Sequence (5' to 3')** | **Reverse Sequence (5' to 3')** | **Size** |
| --- | --- | --- | --- |
| hGAPDH | GTC TCC TCT GAC TTC AAC AGC G | ACC ACC CTG TTG CTG TAG CCA A | 131 |
| hAR | CAG CAG GAA GCA GTA TCC GA | ACA CCG ACA CTG CCT TAC AC | 145 |
| hSLC39A9 | TGG TGA CTG TTT TGG GTG CT | TGC TTG GTG GTG TTT TCC CT | 113 |
| hSRD5A1 | ATG GAG TGG TGT GGC TAT GC | TTT CCG GAG GTA CCA CTC AT | 120 |
| hSRD5A2 | GGG TGG TAC ACA GAC ATA CGG | AGC TGA TTT CTC CAG GCT TCC | 118 |
